# Supplementary material for: Heat adaptation of phage T7 under an extended genetic code
Source: Virus Evol. 2021 Dec 1;7(2):veab100. doi: 10.1093/ve/veab100 (PMC8923235; doi:10.1093/ve/veab100)
Supplement: veab100_Supp [file veab100_supp.zip › Sequencing_Analysis.zip!/Sequencing_Analysis/S3/evidence/JC_49.html]

BRESEQ :: Evidence


| New junction evidence | | | | | | | | | | | |
| --- | --- | --- | --- | --- | --- | --- | --- | --- | --- | --- | --- |
|  | | seq id | position | reads (cov) | reads (cov) | score | skew | freq | annotation | gene | product |
| \* | ? | NC\_001604.1 | 8888 = | 2048 (1.190) | 3 (0.000) | 3/592 | 47.8 | 0.15% | intergenic (–/–) | *–/–* | –/– |
|  | ? | NC\_001604.1 | 8897 = | 2013 (1.190) |  |  |  |  | intergenic (–/–) | *–/–* | –/– |
| Rejected: Coverage evenness skew score above cutoff. | | | | | | | | | | | |
| Rejected: Frequency below/above cutoff threshold. | | | | | | | | | | | |

`ACCCAGCGCAGAGGTGAAAATCTTCTTAGCCATAATGTTAATCTCCTTTAGGTTTCGTTTGGTTTCTCAGAGGGAGTCTTCCCTAATAGTGAGTCGTATTACTTCGGTGCTACACAAGGACGCACACGAGTAACCTCAAAGCCAGCCGGAACGTATTGCCATTCGGCTAACTCCAGAGCTTCGTCTAGGGTCTCAGCGTAGATTGGAACCTCGAAGGAATGCTCCGAGGACTCTACGGTAGCCCAAAACTTCTTATTGTCCACACTAAGTGAACCTGTATTTACGTTTGACATAGTCATTGAC‑‑‑‑‑‑‑‑‑‑‑‑‑‑‑‑‑‑‑‑‑‑‑‑‑‑‑‑‑‑‑‑‑‑‑‑‑‑‑‑‑‑‑‑‑‑‑‑‑‑‑‑‑‑‑‑‑‑‑‑‑‑‑‑‑‑‑‑‑‑‑‑‑‑‑‑‑‑‑‑‑‑‑‑‑‑‑‑‑‑‑‑‑‑‑‑‑‑‑‑‑‑‑‑‑‑‑‑‑‑‑‑‑‑‑‑‑‑‑‑‑‑‑‑‑‑‑‑‑‑‑‑‑‑‑‑‑‑‑‑‑‑‑‑‑‑‑‑‑‑‑‑‑‑‑‑‑‑‑‑‑‑‑‑‑‑‑‑‑‑‑‑‑‑‑‑‑‑‑‑‑‑‑‑‑‑‑‑‑‑‑‑‑‑‑‑‑‑‑‑‑‑‑‑‑‑‑‑‑‑‑‑‑‑‑‑‑‑‑‑‑‑‑‑‑‑‑‑‑‑‑‑‑‑‑‑‑‑‑‑‑‑‑‑‑‑‑‑‑‑‑‑‑‑‑‑‑‑‑‑‑‑‑‑‑‑‑‑‑‑‑‑‑‑‑‑‑‑‑‑‑‑‑‑‑‑‑‑‑‑‑‑‑‑‑‑‑‑‑‑  <  NC_001604.1/9190‑8888`  
`‑‑‑‑‑‑‑‑‑‑‑‑‑‑‑‑‑‑‑‑‑‑‑‑‑‑‑‑‑‑‑‑‑‑‑‑‑‑‑‑‑‑‑‑‑‑‑‑‑‑‑‑‑‑‑‑‑‑‑‑‑‑‑‑‑‑‑‑‑‑‑‑‑‑‑‑‑‑‑‑‑‑‑‑‑‑‑‑‑‑‑‑‑‑‑‑‑‑‑‑‑‑‑‑‑‑‑‑‑‑‑‑‑‑‑‑‑‑‑‑‑‑‑‑‑‑‑‑‑‑‑‑‑‑‑‑‑‑‑‑‑‑‑‑‑‑‑‑‑‑‑‑‑‑‑‑‑‑‑‑‑‑‑‑‑‑‑‑‑‑‑‑‑‑‑‑‑‑‑‑‑‑‑‑‑‑‑‑‑‑‑‑‑‑‑‑‑‑‑‑‑‑‑‑‑‑‑‑‑‑‑‑‑‑‑‑‑‑‑‑‑‑‑‑‑‑‑‑‑‑‑‑‑‑‑‑‑‑‑‑‑‑‑‑‑‑‑‑‑‑‑‑‑‑‑‑‑‑‑‑‑‑‑‑‑‑‑‑‑‑‑‑‑‑‑‑‑‑‑‑‑‑‑‑‑‑‑‑‑‑‑‑‑‑‑‑‑‑‑tgacTATGTCAAACGTAAATACAGGTTCACTTAGTGTGGACAATAAGAAGTTTTGGGCTACCGTAGAGTCCTCGGAGCATTCCTTCGAGGTTCCAATCTACGCTGAGACCCTAGACGAAGCTCTGGAGTTAGCCGAATGGCAATACGTTCCGGCTGGCTTTGAGGTTACTCGTGTGCGTCCTTGTGTAGCACCGAAGTAATACGACTCACTATTAGGGAAGACTCCCTCTGAGAAACCAAACGAAACCTAAAGGAGATTAACATTATGGCTAAGAAGATTTTCACCTCTGCGCTGGGTACCGCT  >  NC_001604.1/8897‑9196`  

                                                                                                                                                                                                                                                                                                                                                                                                                                                                                                                                                                                                                             
`ACCCAGCGCAGAGGTGAAAATCTTCTTAGCCATAATGTTAATCTCCTTTAGGTTTCGTTTGGTTTCTCAGAGGTAGTCTTCCCTAATAGTGAGTCGTATTACTTCGGTGCTACACAAGGACGCACACGAGTAACCTCAAAGCCAGCCGGAACGTATTGCCATTCGGCTAACTCCAGAGCTTCGTCTAGGGTCTCAGCGTAGATTGGAACCTCGAAGGAATGCTCCGAGGACTCTACGGTAGCCCAAAACTTCTTATTGTCCACACTAAGTGAACCTGTATTTACGTTTGACATAGTCATTG                                                                                                                                                                                                                                                                                                                >  1:218230/1‑301`  
`ACCCAGCGCAGAGGTGAAAATCTTCTTAGCCATAATGTTAATCTCCTTTAGGTTTCGTTTGGTTTCTCAGAGGGAGTCTTCCCTAATAGTGAGTCGTATTACTTCGGTGCTACACAAGGACGCACACGAGTAACCTCAACGCCAGCCGGAACGTATTGCCATTCGGCTAACTCCAGAGCTTCGTCTAGGGTCTCAGCGTAGATTGGAACCTCGAAGGAATGCTCCGAGGACTCTACGGTCGCCCAAAACTTCTTATTGTCCACACTAAGTGAACCTGTATTTACGTTTGACATAGTCATTt                                                                                                                                                                                                                                                                                                                >  1:1238/1‑300`  
`ACCCAGCGCAGAGGTGAAAATCTTCTTAGCCATAATGTTAATCTCCTTTAGGTTTCGTTTGGTTTCTCAGAGGGAGTCTTCCCTAATAGTGAGTCGTATTACTTCGGTGCTACACAAGGACGCACACGAGTAACCTCAAAGCCAGCCGGAACGTATTGCCATTCGGCTAACTCCAGAGCTTCGTCTATGGTCTCAGCGTAGATTGGAACCTCGAAGGAATGCTCCGAGGACTCTACGGTAGCCCAAAACTTCTTATTGTCCACACTAAGTGAACCTGTATTTACGTTTGACATAGTCATTG                                                                                                                                                                                                                                                                                                                >  1:156958/1‑301`  
`ACCCAGCGCAGAGGTGAAAATCTTCTTAGCCATAATGTTAATCTCCTTTAGGTTTCGTTTGGTTTCTCAGAGGGAGTCTTCCCTAATAGTGAGTCGTATTACTTCGGTGCTACACAAGGACGCACACGAGTAACCTCAAAGCCAGCCGGAACGTATTGCCATTCGGCTAACTCCAGAGCTTCGTCTAGGGTCTCAGCGTAGATTGGAACCTCGAAGGAATGCTCCTAGGACTCTACGGTAGCCCAAAACTTCTTATTGTCCACACTAAGTGAACCTGTATTTACGTTTGACATAGTCATTt                                                                                                                                                                                                                                                                                                                >  1:8924/1‑300`  
`ACCCAGCGCAGAGGTGAAAATCTTCTTAGCCATAATGTTAATCTCCTTTAGGTTTCGTTTGGTTTCTCAGAGGGAGTCTTCCCTAATAGTGAGTCGTATTACTTCGGTGCTACACAAGGACGCACACGAGTAACCTCAAAGCCAGCCGGAACGTATTGCCATTCGGCTAACTCCAGAGCTTCGTCTAGGGTCTCAGCGTAGATTGGAACCTCGAAGGAATGCTCCGAGGACTCTACGGTAGCCCAAAACTTCTTATTGTCCACACTAAGTGAACCTGTATTTACGTTTGACATAGTCATTt                                                                                                                                                                                                                                                                                                                >  1:270475/1‑300`  
`ACCCAGCGCAGAGGTGAAAATCTTCTTAGCCATAATGTTAATCTCCTTTAGGTTTCGTTTGGTTTCTCAGAGGGAGTCTTCCCTAATAGTGAGTCGTATTACTTCGGTGCTACACAAGGACGCACACGAGTAACCTCAAAGCCAGCCGGAACGTATTGCCATTCGGCTAACTCCAGAGCTTCGTCTAGGGTCTCAGCGTAGATTGGAACCTCGAAGGAATGCTCCGAGGACTCTACGGTAGCCCAAAACTTCTTATTGTCCACACTAAGTGAACCTGTATTTACGTTTGACATAGTCATTG                                                                                                                                                                                                                                                                                                                <  1:98578/301‑1`  
`ACCCAGCGCAGAGGTGAAAATCTTCTTAGCCATAATGTTAATCTCCTTTAGGTTTCGTTTGGTTTCTCAGAGGGAGTCTTCCCTAATAGTGAGTCGTATTACTTCGGTGCTACACAAGGACGCACACGAGTAACCTCAAAGCCAGCCGGAACGTATTGCCATTCGGCTAACTCCAGAGCTTCGTCTAGGGTCTCAGCGTAGATTGGAACCTCGAAGGAATGCTCCGAGGACTCTACGGTAGCCCAAAACTTCTTATTGTCCACACTAAGTGAACCTGTATTTACGTTTGACATAGTCATTG                                                                                                                                                                                                                                                                                                                >  1:199795/1‑301`  
`ACCCAGCGCAGAGGTGAAAATCTTCTTAGCCATAATGTTAATCTCCTGTAGGTTTCGTTTGGTTTCTCAGAGGGAGTCTTCCCTAATAGTGAGTCGTATTACTTCGGTGCTACACAAGGACGCACACGAGTAACCTCAAAGCCAGCCGGAACGTATTGCCATTCGGCTAACTCCAGAGCTTCGTCTAGGGTCTCAGCGTAGATTGGAACCTCGAAGGAATGCTCCGAGGACTCTACGGTAGCCCAAAACTTCTTATTGTCCACACTAAGTGAACCTGTATTTACGTTTGACATAGTCATTG                                                                                                                                                                                                                                                                                                                <  1:168291/301‑1`  
`ACCCAGCGCAGAGGTGAAAATCTTCTTAGCCATAATGTTAATCTCCGTTAGGTTTCGTTTGGTTTCTCAGAGGGAGTCTTCCCTAATAGTGAGTCGTATTACTTCGGTGCTACACAAGGACGCACACGAGTAACCTCAAAGCCAGCCGGAACGTATTGCCATTCGGCTAACTCCAGAGCTTCGTCTAGGGTCTCAGCGTAGATTGGAACCTCGAAGGAATGCTCCGAGGACTCTACGGTAGCACAAAACTTCTTATTGTCCACACTAAGTGAACCTGTATTTACGTTTGACATAGTCATTG                                                                                                                                                                                                                                                                                                                >  1:151408/1‑301`  
`ACCCAGCGCAGAGGTGAAAATCTTCTTAGCCATAATGATAATCTCCTTTAGGTTTCGTTTGGTTTCTCAGAGGGAGTCTTCCCTAATAGTGAGTCGTATTACTTCGGTGCTACACAAGGACGCACACGAGTAACCTCAAAGCCAGCCGGAACGTATTGCCATTCGGCTAACTCCAGAGCTTCGTCTAGGGTCTCAGCGTAGATTGGAACCTCGAAGGAATGCTCCGAGGACTCTACGGTAGCCCAAAACTTCTTATTGTCCACACTAATTGAACCTGTATTTCCGTTTGACATAGTCATTG                                                                                                                                                                                                                                                                                                                >  1:264002/1‑301`  
`ACCCAGCGCAGAGGTGAAAATCTTCTGAGCCATAATGTTAATCTCCTTTAGGTTTCGTTTGGTTTCTCAGAGGGAGTCTTCCCTAATAGTGAGTCGTATTACTTCGGTGCTACACAAGGACGCACACGAGTAACCTCAAAGCCAGCCGGAACGTATTGCCATTCGGCTAACTCCAGAGCTTCGTCTAGGGTCTCAGCGTAGATTGGAACCTCGAAGGAATGCTCCGAGGACTCTACGGTAGCCCAAAACTTCTTATTGTCCACACTAAGTGAACCTGTATTTACGTTTGACATAGTCATTG                                                                                                                                                                                                                                                                                                                <  1:100077/301‑1`  
 `CCCCGCGCAGAGGTGAAAATCTTCTTAGCCATAATGTTAATCTCCTTTAGGTTTCGTTTGGTTTCTCAGAGGGAGTCTTCCCTAATAGTGAGTCGTATTACTTCGGTGCTACACAAGGACGCACACGAGTAACCTCAAAGCCAGCCGGAACGTATTGCCATTCGGCTAACTCCAGAGCTTCGTCTAGGGTCTCAGCGTAGATTGGAACCTCGAAGGAATGCTCCGAGGACTCTACGGTAGCCCAAAACTTCTTATTGTCCACACTAAGTGAACCTGTATTTACGTTTGACATAGTCATTGA                                                                                                                                                                                                                                                                                                               >  1:131158/1‑301`  
  `ggctcggagatgtgtataagagacagGCCATAATGTTAATCTCCTTTAGGTTTCGTTTGGTTTCTCAGAGGGAGTCTTCCCTAATAGTGAGTCGTATTACTTCGGTGCTACACAAGGGCGCACACGAGTAACCTCAAAGCCAGCCGGAACGTATTGCCATTCGGCTAACTCCAGAGCTTCGTCTAGGGTCTCAGCGTAGATTGGAACCTCGAAGGAATGCTCCGAGGACTCTACGGTAGCCCAAAACTTCTTATTGTCCACACTAAGTGAACCTGTATTTACGTTTGACATAGTCATTGAC                                                                                                                                                                                                                                                                                                              <  1:191227/275‑1`  
  `cggagatgtgtataagagacagCTTAGCCATAATGTTAATCTCCTTTAGGTTTCGTTTGGTTTCTCAGAGGGAGTCTTCCCTAATAGTGAGTCGTATTACTTCGGTGCTACACAAGGATGCACACGAGTAACCTCAAAGCCAGCCGGAACGTATTGCCATTCGGCTAACTCCAGAGCTTCGTCTAGGGTCTCAGCGTAGATTGGAACCTCGAAGGAATGCTCCGATGACTCTACGGTAGCCCAAAACTTCTTATTGTCCACACTAAGTGAACCTGTATTTACGTTTGACATAGTCATTGAC                                                                                                                                                                                                                                                                                                              <  1:299619/279‑1`  
  `cccGCGCAGAGGTGAAAATCTTCTTAGCCATAATGTTAATCTCCTTTAGGTTTCGTTTGGTTTCTCAGAGGGAGTCTTCCCTAATAGTGAGTCGTATTACTTCGGTGCTACACAAGGACGCACACGAGTAACCTCAAAGCCAGCCGGAACGTATTGCCATTCGGCTAACTCCAGAGCTTCGTCTAGGGTCTCAGCGTAGATTGGAACCTCGAAGGAATGCTCCGAGGACTCTACGGTAGCCCAAAACTTCTTATTGTCCACACTAAGTGAACCTGTATTTACGTTTGACATAGTCATTGAC                                                                                                                                                                                                                                                                                                              <  1:129020/298‑1`  
  `cagGCGCAGAGGTGAAAATCTTCTTAGCCATAATGTTAATCTCCTTTAGGTTTCGTTTGGTTTCTCAGAGGGAGTCTTCCCTAATAGTGAGTCGTATTACTTCGGTGCTACACAAGGACGCACACGAGTAACCTCAAAGCCAGCCGGAACGTATTGCCATTCGGCTAACTCCAGAGCTTCGTCTAGGGTCTCAGCGTAGATTGGAACCTCGAAGGAATGCTCCGAGGACTCTACGGTAGCCCAAAACTTCTTATTGTCCACACTAAGTGAACCTGTATTTACGTTTGACATAGTCATTGAC                                                                                                                                                                                                                                                                                                              <  1:245834/298‑1`  
  `cagGCGCAGAGGTGAAAATCTTCTTAGCCATAATGTTAATCTCCTTTAGGTTTCGTTTGGTTTCTCAGAGGGAGTCTTCCCTAATAGTGAGTCGTATTACTTCGGTGCTACACAAGGACGCACACGAGTAACCTCAAAGCCAGCCGGAACGTATTGCCATTCGGCTAACTCCAGAGCTTCGTCTAGGGTCTCAGCGTAGATTGGAACCTCGAAGGAATGCTCCGAGGACTCTACGGTAGCCCAAAACTTCTTATTGTCCACACTAAGTGAACCTGTATTTACGTTTGACATAGTCATTGAC                                                                                                                                                                                                                                                                                                              <  1:155012/298‑1`  
  `caAGCGCAGAGGTGAAAATCTTCTTAGCCATAATGTTAATCTCCTTTAGGTTTCGTTTGGTTTCTCAGAGGGAGTCTTCCCTAATAGTGAGTCGTATTACTTCGGTGCTACACAAGGACGCACACGAGTAACCTCAAAGCCAGCCGGAACGTATTGCCATTCGGCTAACTCCAGAGCTTCGTCTAGGGTCTCAGCGTAGATTGGAACCTCGAAGGAATGCTCCGAGGACTCTACGGTAGCCCAAAACTTCTTATTGTCCACACTAAGTGAACCTGTATTTACGTTTGACATAGTCATTGAC                                                                                                                                                                                                                                                                                                              <  1:251348/299‑1`  
  `agatgtgtataagagacagCTTCTTAGCCATAATGTTAATCTCCTTTAGGTTTCGTTTGGTTTCTCAGAGGGAGTCTTCCCTAATAGTGAGTCGTATTACTTCGGTGCTACACAAGGACGCACACGAGTAACCTCAAAGCCAGCCGGAACGTATTGCCATTCGGCTAACTCCAGAGCTTCGTCTAGGGTCTCAGCGTAGATTGGAACCTCGAAGGAATGCTCCGAGGACTCTACGGTAGCCCAAAACTTCTTATTGTCCACACTAAGTGAACCTGTATTTACGTTTGACATAGTCATTGAC                                                                                                                                                                                                                                                                                                              <  1:48262/282‑1`  
  `agagacagGAGGTGAAAATCTTCTTAGCCATAATGTTAATCTCCTTTAGGTTTCGTTTGGTTTCTCAGAGGGAGTCTTCCCTAATAGTGAGTCGTATTACTTCGGTGCTACACAAGGACGCACACGAGTAACCTCAAAGCCAGCCGGAACGTATTGCCATTCGGCTAACTCCAGAGCTTCGTCTAGGGTCTCAGCGTAGATTGGAACCTCGAAGGAATGCTCCGAGGACTCTACGGTAGCCCAAAACTTCTTATTGTCCACACTAAGTGAACCTGTATTTACGTTTGACATAGTCATTGAC                                                                                                                                                                                                                                                                                                              <  1:217393/293‑1`  
  `CCATCGTAGAAGTGAAAATCATTTTACCTTTACTGTTGATCTTCGTTACGTTTCGTTTGGTATCTCAGTGGGAGTCTTCCCTAATAGTGAGTTGTATTACTTCGTTGCTACTCAAGGTCGCATACGAGTAACCTCAAAGCCAGCCGGAACGTATTGCCATTCGGCTAACTCCTGAGCTTCGTCTAGGGTCTCAGCTCAGATTGGAACCTCGAAGGAATGCTCCGAGGACTCTACGGTAGCCCAAAACTTCTTATTGTCCACACTAAGTGAACCTGTATTTACGTTTGACATAGTCATTGAC                                                                                                                                                                                                                                                                                                              <  1:233039/301‑1`  
  `CCAGCGCAGAGTTGAAAATCTGCTTAGCCATAATGTTAATCGCCTTTAGGTTTCGTTTGGTTTCTCAGAGGGAGTCTTCCCTAATAGGGAGTCGTATTACTTCGGTGCTACACAAGGACGCACACGAGTAACCTCAAAGCCAGCCGGAACGTATTGCCATTCGGCTAACTCCAGAGCTTCGTCTAGGGTCTCAGCGTAGATTGGAACCTCGAAGGAATGCTCCGAGGACTCTACGGTAGCCCAAAACTTCTTATTGTCCACACTAAGTGAACCTGTATTTACGTTTGACATAGTCATTGAC                                                                                                                                                                                                                                                                                                              <  1:93684/301‑1`  
  `CCAGCGCAGAGGTTAAAATCTTCTTAGCCATAATGTTAATCTCCTTTAGGTTTCGTTTGGTTTCTCAGAGGGAGTCTTCCCTAATAGTGAGTTGTATTACTTCGGTGCTACACAAGGACGCACACGAGTAACCTCAAAGCCAGCCGGAACGTATTGCCATTCGGCTAACTCCAGAGCTTCGTCTAGGGTCTCAGCGTAGATTGGAACCTCGAAGGAATGCTCCGAGGACTCTACGGTAGCCCAAAACTTCTTATTGTCCACACTAAGTGAACCTGTATTTACGTTTGACATAGTCATTGAC                                                                                                                                                                                                                                                                                                              <  1:231985/301‑1`  
  `CCAGCGCAGAGGTGAAAATCTTCTTAGCCTTAATGTTAATCTCCTTTAGGTTTCGTTTGGTTTCTCAGAGGGAGTCTTCCCTAATAGTGAGTCGTATTACTTCGGTGCTACACAAGGACGCACACGAGTAACCTCAAAGCCAGCCGGAACGTATTGCCATTCGGCTAACTCCAGAGCTTCGTCTAGGGTCTCAGCGTAGATTGGAACCTCGAAGGAATGCTCCGAGGACTCTACGGTAGCCCAAAACTTCTTATTGTCCACACTAAGTGAACCTGTATTTACGTTTGACATAGTCATTGAC                                                                                                                                                                                                                                                                                                              <  1:127099/301‑1`  
  `CCAGCGCAGAGGTGAAAATCTTCTTAGCCATAATGTTAATCTCCTTTAGTTTTCGTTTGGTTTCTCAGAGGGAGTCTTCCCTAATAGTGAGTCGTATTACTTCGTTGCTACACAAGGACGCACACGAGTAACCCCAAAGCCAGCCGGAACGTATTGCCATTCGGCTAACTCCAGAGCTTCGTCTAGGGTCTCAGCGTAGATTGGAACCTCGAAGGAATGCTCCTAGGACTCTACGGTAGCCCAAAACTTCTTATTGTCCACACTAAGTGAACCTGTATTTACGTTTGACATAGTCATTGAC                                                                                                                                                                                                                                                                                                              <  1:301158/301‑1`  
  `CCAGCGCAGAGGTGAAAATCTTCTTAGCCATAATGTTAATCTCCTTTAGGTTTCGTTTTGTTTCTCAGAGGGAGTCTTCCCTAATAGTGAGTCGTATTACTTCGGTGCTACACAAGGACGCACACGAGTAACCTCAAAGCCAGCCGGAACGTATTGCCATTCGGCTAACTCCAGAGCTTCGTCTAGGGTCTCAGCGTAGATTGGAACCTCGAAGGAATGCTCCGAGGACTCTACGGTAGCCCAAAACTTCTTATTGTCCACACTAAGTGAACCTGTATTTACGTTTGACATAGTCATTGAC                                                                                                                                                                                                                                                                                                              <  1:263000/301‑1`  
  `CCAGCGCAGAGGTGAAAATCTTCTTAGCCATAATGTTAATCTCCTTTAGGTTTCGTTTGGTTTCTCAGAGGGAGTCTTCCCTAATAGTGAGTCGTATTACTTCGGTGCTACACAAGGACGCACACGAGTAACCTCAAAGCCAGCCGGAACGTATTGCCATTCGGCTAACTCCAGAGCTTCGTCTAGGGTCTCAGCGTAGATTGGAACCTCGAAGGAATGCTCCGAGGACTCTACGGTAGCCCAAAACTTCTTATTGTCCACACTAAGTGAACCTGTATTTACGTTTGACATAGTCATTGAC                                                                                                                                                                                                                                                                                                              <  1:283322/301‑1`  
  `CCAGCGCAGAGGTGAAAATCTTCTTAGCCATAATGTTAATCTCCTTTAGGTTTCGTTTGGTTTCTCAGAGGGAGTCTTCCCTAATAGTGAGTCGTATTACTTCGGTGCTACACAAGGACGCACACGAGTAACCTCAAAGCCAGCCGGAACGTATTGCCATTCGGCTAACTCCAGAGCTTCGTCTAGGGTCTCAGCGTAGATTGGAACCTCGAAGGAATGCTCCGAGGACTCTACGGTAGCCCAAAACTTCTTATTGTCCACACTAAGTGAACCTGTATTTACGTTTGACATAGTCATTGAC                                                                                                                                                                                                                                                                                                              <  1:285018/301‑1`  
  `CCAGCGCAGAGGTGAAAATCTTCTTAGCCATAATGTTAATCTCCTTTAGGTTTCGTTTGGTTTCTCAGAGGGAGTCTTCCCTAATAGTGAGTCGTATTACTTCGGTGCTACACAAGGACGCACACGAGTAACCTCAAAGCCAGCCGGAACGTATTGCCATTCGGCTAACTCCAGAGCTTCGTCTAGGGTCTCAGCGTAGATTGGAACCTCGAAGGAATGCTCCGAGGACTCTACGGTAGCCCAAAACTTCTTATTGTCCACACTAAGTGAACCTGTATTTACGTTTGACATAGTCATTGAC                                                                                                                                                                                                                                                                                                              <  1:114959/301‑1`  
  `CCAGCGCAGAGGTGAAAATCTTCTTAGCCATAATGTTAATCTCCTTTAGGTTTCGTTTGGTTTCTCAGAGGGAGTCTTCCCTAATAGTGAGTCGTATTACTTCGGTGCTACACAAGGACGCACACGAGTAACCTCAAAGCCAGCCGGAACGTATTGCCATTCGGCTAACTCCAGAGCTTCGTCTAGGGTCTCAGCGTAGATTGGAACCTCGAAGGAATGCTCCGAGGACTCTACGGTAGCCCAAAACTTCTTATTGTCCACACTAAGTGAACCTGTATTTACGTTTGACATAGTCATTGAC                                                                                                                                                                                                                                                                                                              <  1:81134/301‑1`  
  `CCAGCGCAGAGGTGAAAATCTTCTTAGCCATAATGTTAATCTCCTTTAGGTTTCGTTTGGTTTCTCAGAGGGAGTCTTCCCTAATAGTGAGTCGTATTACTTCGGTGCTACACAAGGACGCACACGAGTAACCTCAAAGCCAGCCGGAACGTATTGCCATTCGGCTAACTCCAGAGCTTCGTCTAGGGTCTCAGCGTAGATTGGAACCTCGAAGGAATGCTCCGAGGACTCTACGGTAGCCCAAAACTTCTTATTGTCCACACTAAGTGAACCTGTATTTACGTTTGACATAGTCATTGAC                                                                                                                                                                                                                                                                                                              <  1:18146/301‑1`  
  `CCAGCGCAGAGGTGAAAATCTTCTTAGCCATAATGTTAATCTCCTTTAGGTTTCGTTTGGTTTCTCAGAGGGAGTCTTCCCTAATAGTGAGTCGTATTACTTCGGTGCTACACAAGGACGCACACGAGTAACCTCAAAGCCAGCCGGAACGTATTGCCATTCGGCTAACTCCAGAGCTTCGTCTAGGGTCTCAGCGTAGATTGGAACCTCGAAGGAATGCTCCGAGGACTCTACGGTAGCCCAAAACTTCTTATTGTCCACACTAAGTGAACCTGTATTTACGTTTGACATAGTCATTGAC                                                                                                                                                                                                                                                                                                              <  1:257502/301‑1`  
  `CCAGCGCAGAGGTGAAAATCTTCTTAGCCATAATGTTAATCTCCTTTAGGTTTCGTTTGGTTTCTCAGAGGGAGTCTTCCCTAATAGTGAGTCGTATTACTTCGGTGCTACACAAGGACGCACACGAGTAACCTCAAAGCCAGCCGGAACGTATTGCCATTCGGCTAACTCCAGAGCTTCGTCTAGGGTCTCAGCGTAGATTGGAACCTCGAAGGAATGCTCCGAGGACTCTACGGTAGCCCAAAACTTCTTATTGTCCACACTAAGTGAACCTGTATTTACGTTTGACATAGTCATTGAC                                                                                                                                                                                                                                                                                                              <  1:81348/301‑1`  
  `CCAGCGCAGAGGTGAAAATCTTCTTAGCCATAATGTTAATCTCCTTTAGGTTTCGTTTGGTTTCTCAGAGGGAGTCTTCCCTAATAGTGAGTCGTATTACTTCGGTGCTACACAAGGACGCACACGAGTAACCTCAAAGCCAGCCGGAACGTATTGCCATTCGGCTAACTCCAGAGCTTCGTCTAGGGTCTCAGCGTAGATTGGAACCTCGAAGGAATGCTCCGAGGACTCTACGGTAGCCCAAAACTTCTTATTGTCCACACTAAGTGAACCTGTATTTACGTTTGACATAGTCATTGAC                                                                                                                                                                                                                                                                                                              <  1:336538/301‑1`  
  `CCAGCGCAGAGGTGAAAATCTTCTTAGCCATAATGTTAATCTCCTTTAGGTTTCGTTTGGTTTCTCAGAGGGAGTCTTCCCTAATAGTGAGTCGTATTACTTCGGTGCTACACAAGGACGCACACGAGTAACCTCAAAGCCAGCCGGAACGTATTGCCATTCGGCTAACTCCAGAGCTTCGTCTAGGGTCTCAGCGTAGATTGGAACCTCGAAGGAATGCTCCGAGGACTCTACGGTAGCCCAAAACTTCTTATTGTACACACTAAGTGAACCTGTATTTACGTTTGACATAGTCATTGAC                                                                                                                                                                                                                                                                                                              <  1:227332/301‑1`  
  `CCAGCGCAGAGGTGAAAATCTTCTTAGCCATAATGTTAATCTCCTTTAGGTTTCGGTTGGTTTCTCAGAGGGAGTCTTCCCTAATAGTGAGTCGTATTACTTCGGTGCTACACAAGGACGCACACGAGTAACCTCAAAGCCAGCCGGAACGTATTGCCATTCGGCTAACTCCAGAGCTTCGTCTAGGGTCTCAGCGTAGATTGGAACCTCGAAGGAATGCTCCGAGGACTCTACGGTAGCCCAAAACTTCTTATTGTCCACACTAAGTGAACCTGTATTTACGTTTGACATAGTCATTGAC                                                                                                                                                                                                                                                                                                              <  1:127576/301‑1`  
  `CCAGCGCAGAGGTGAAAATCTTCTTAGCCATAATGTTAATCTACTTTAGGTTTCGTTTGGTTTCTCAGAGGGAGTCTTCCCTAATAGTGAGTCGTATTACTTCGGTGCTACACAAGGACGCACACGAGTAACCTCAAAGCCAGCCGGAACGTATTGCCATTCGGCTAACTCCAGAGCTTCGTCTAGGGTCTCAGCGTAGATTGGAACCTCGAAGGAATGCTCCGAGGACTCTACGGTAGCCCAAAACTTCTTATTGTCCACACTAAGTGAACCTGTATTTACGTTTGACATAGTCATTGAC                                                                                                                                                                                                                                                                                                              <  1:308561/301‑1`  
  `CCAGCGCAGAGGTGAAAATCTTCTTAGCCATAATGTTAATCGCAGTTAGGTTTCGTTTGGTTTCTCAGAGGGAGTCTTACCTAATAGTGAGTCGTATTACGTAGGTGCTACACAAGGACGCACACGAGGAACGTCAAAGCCAGCCGGAACGTATTGCCATTCGGCTAACTCCAGAGCTTCGTCTAGGGTCTCAGCGTAGATTGGAACCTCGAAGGAATGCTCCGAGGACTCTACGGTAGCCCAAAACTTCTTATTGTCCACACTAAGTGAACCTGTATTTACGTTTGACATAGTCATTGAC                                                                                                                                                                                                                                                                                                              <  1:337148/301‑1`  
  `CCAGCGCAGAGGTGAAAATCTTCTTAGCCAGAATGTTAATCTCCTTTAGGTGTCGGTTGGTTTCTCAGAGGGAGTCTTCCCTAATAGTGAGTCGTATTACTTCGGTGCTACACAAGGACGCACACGAGTAACCTCAAAGCCAGCCGGAACGTATTGCCATTCGGCTAACTCCAGAGCTTCGTCTAGGGTCTCAGCGTAGATTGGAACCTCGAAGGAATGCTACGAGGACTCTACGGTAGCCCAAAACTTCTTATTGTCCACACTAAGTGAACCTGTATTTACGTTTGACATAGTCATTGAC                                                                                                                                                                                                                                                                                                              <  1:31060/301‑1`  
  `CCAGCGCAGAAGTGAAAATCTTCTTAGCCATAATGTTAGTCTCCTTTAGGTTTCGTTTGGTTTCTCAGAGGGAGTCTTCCCTAATAGTGAGTCGTATTACTTCGGTGCTACACAAGGACGCACACGAGTAACCTCAAAGCCAGCCGGAACGTATTGCCATTCGGCTAACTCCAGAGCTTCGTCTAGGGTCTCAGCGTAGATTGGAACCTCGAAGGAATGCTCCGAGGACTCTACGGTAGCCCAAAACTTCTTATTGTCCACACTAAGTGAACCTGTATTTACGTTTGACATAGTCATTGAC                                                                                                                                                                                                                                                                                                              <  1:284337/301‑1`  
  `CCAGAGAAGAGGTGAAAATCTTCTTAGCCATAATGTTAATCTCCTTTAGGTTTCGTTTGGTTTCTCAGAGGGAGTCTTCCCTAATAGTGAGTCGTATTACTTCGGTGCTACACAAGGACGCACACGAGTAACCTCAAAGCCAGCCGGAACGTATTGCCATTCGGCTAACTCCAGAGCTTCGTCTAGGGTCTCAGCGTAGATTGGAACCTCGAAGGAATGCTCCGAGGACTCTACGGTAGCCCAAAACTTCTTATTGTCCACACTAAGTGAACCTGTATTTACGTTTGACATAGTCATTGAC                                                                                                                                                                                                                                                                                                              <  1:10703/301‑1`  
     `GCGCAGAGGTGAAAATCTTCTTAGCCATAATGTTAATCTCCTTTAGGGTTCGTTTGGTTTCTCAGAGGGAGTCTTCCCTAATAGTGAGTCGTATTACTTCGGTGCTACACAAGGACGAGCACGAGTAACCTCAAAGCAAGCCGGAACGTATTGCCATTCGGCTAACTCCGGAGCTTCGTCTAGGGTCTCAGCGTAGATTGGAACCTCGAAGGAATGCTCCGAGGACGCTACGGTAGCCCAAAACTTCTTATTGTCCACACTAATTGAACCGGTATTTAAGTTTGACATAGTCATTGACatc                                                                                                                                                                                                                                                                                                           >  1:147642/1‑298`  
                                                                `tcgCAGAGGGAGTCTTCCCTAATGGTGAGTCCTATTAATTCGGTGCTACACAAGGACGCTCACGAGTACCCTCAAAGCCAGCCGGAACGTATTGCCATTTGGCTAACTCCAGAGCTTCGTCTAGGGTCTCAGCGTAGATTGGAACCTCGAAGGAATGCTCCGAGGACTCTACGGTAGCCCAAAACTTCTTAGTGTCCACACTAAGTGAACCTGTATTTACGTTTGACATAGTCATTGACTATGTCAAACGTAAATACAGGTTCACTTAGTGTGGACAATAAGAAGTTTTGGGCTACCGTAG                                                                                                                                                                                                                                                <  1:320868/298‑1`  
                                                                                       `AGTGAGTCGTATTACTTCGGTGCTACACAAGGACGCACACGAGTAACCTCAAAGCCAGCCGGAACGTATTGCCATTCGGCTAACTCCAGAGCTTCGTCTAGGGTCTCAGCGTAGATTGGAACCTCGAAGGAATGCTCCGAGGACTCTACGGTAGCCCAAAACTTCTTATTGTCCACACTAAGTGAACCTGTATTTACGTTTGACATAGTCATTGACTATGTCAAACGTAAATACAGGTTCACTTAGTGTGGACAATAAGAAGTTTTGTGCTACCGTAGAGTCCTCTGActgtcccttataa                                                                                                                                                                                                                         >  1:154990/1‑288`  
                                                                                            `GTCGTATTACTTCGGTGCTACACAAGGACGCACACGAGTAACCTCAAAGCCAGCCGGAACGTATTGCCATTCGGCTAACTCCAGAGCTTCGTCTAGGGTCTCAGCGTAGATTGGAACCTCGAAGGAATGCTCCGAGGACTCTACGGTAGCCCAAAACTTCTTATTGTCCACACTAAGTGAACCTGTATTTACGTTTGACATAGTCATTGACTATGTCAAACGTAAATACAGGTTCACTTAGTGTGGACAATAAGAAGTTTTGGGCTACCGTAGAGTCCTgactcttatacacatctccgag                                                                                                                                                                                                                    >  1:328071/1‑279`  
                                                                                                                                                                                                                                                                                                          `caGACTATTTCAAACGTAAATACAGGTTCACTTAGTGTGGACAATAAGAATTTTTGGTCTACCTTAGAGTCCTCGGAGCATTCCTTCGAGTTTCCAATCTACGCTGAGACCCTAGACGAAGCTCTGGAGTTAGCCGAATGGCAATACGTTCCGTCTGGCTTTGAGGTTACTCGTGTGCGTCCTTGTGTAGCACCGAAGTAATACGACTCACTATTAGGGAATACTCCCTCTGAGAAACCAAACGAAACCTAAAGGAGATTAACATTATTGCTAAGAAGATTTTCACCTCTGCGCTGGGTAC      <  1:56625/299‑1`  
                                                                                                                                                                                                                                                                                                           `TGACTATGTCAAACGTAAATACAGGTTCACTTAGTGTGGACAATAAGAAGTTTTGGGCTACCGTAGAGTCCTCGGAGCATTCCTTCGAGGTTCCAATCTACGCTGAGACCCTAGACGAAGCTCTGGAGTTAGCCGAATGGCAATACGTTCCGGCTGGCTTTGAGGTTACTCGTGTGCGTCCTTGTGTAGCACCGAAGTAATACGACTCACTATTAGGGAAGACTCCCTCTGAGAAACCAAACGAAACCTAAAGGAGATTAACATTATGGCTAAGAAGATTTTCACCTCTGCGCTGGGTACC     >  1:188706/1‑301`  
                                                                                                                                                                                                                                                                                                           `TGACTATGTCAAACGTAAATACAGGTTCACTTAGTGTGGACAATAAGAAGTTTTGGGCTACCGTAGAGTCCTCGGAGCATTCCTTCGAGGTTCCAATCTACGCTGAGACCCTAGACGAAGCTCTGGAGTTAGCCGAATGGCAATACGTTCCGGCTGGCTTTGAGGTTACTCGTGTGCGTCCTTGTGTAGCACCGAAGTAATACGACTCACTATTAGGGAAGACTCCCTCTGAGAAACCAAACGAAACCTAAAGGAGATTAACATTATGGCTAAGAAGATTTTCACCTCTGCGCTGGGTACC     >  1:12989/1‑301`  
                                                                                                                                                                                                                                                                                                           `TGACTATGTCAAACGTAAATACAGGTTCACTTAGTGTGGACAATAAGAAGTTTTGGGCTACCGTAGAGTCCTCGGAGCATTCCTTCGAGGTTCCAATCTACGCTGAGACCCTAGACGAAGCTCTGGAGTTAGCCGAATGGCAATACGTTCCGGCTGGCTTTGAGGTTACTCGTGTGCGTCCTTGTGTAGCACCGAAGTAATACGACTCACTATTAGGGAAGACTCCCTCTGAGAAACCAAACGAAACCTAAAGGAGATTAACATTATGGCTAAGAAGATTTTCACCTCTGCGCTGGGTACC     >  1:313615/1‑301`  
                                                                                                                                                                                                                                                                                                            `GACTATGTCAAACGTAAATACAGGTTCACTTAGTGTGGACAATCAGAAGTTTTGGGCTACCGTAGAGTCCTCGGAGCATTCCTTCGAGGTTCCAATCTACTCTGAGACCCTAGACGAAGCTCTGGAGTTAGCCGAATGGCAATACGTTCCGGCTGGCTTTGAGGTTACTCGTGTGCGTCCTTGTGTAGCACCGAAGTAATACGACTCACTATTAGGGAAGACTCCCTCTGAGAAACCAAACGAAACCTATCGGAGATTAACATTATGGCTAAGAAGATTTTCACATCTGCGCTGGGTACCc    >  1:49168/1‑300`  
                                                                                                                                                                                                                                                                                                            `GACTATGTCAAACGTAAATACAGGTTCACTTAGTGTGGACAATAAGAAGTTTTGGGCTACCGTAGAGTCCTCGGAGCATTCCTTCGAGGTTCCAATCTACGCTGAGACCCTAGACGAAGCTCTGGAGTTAGCCGAATGGCAATACGTTCCGGCTGGCTTTGAGGTTACTCGTGTGCGTCCTTGTGTAGCACCGAAGTAATACGACTCACTATTAGGGAATACTCCCTCTTAGAAACCAAACGAAACCTAAAGGAGATCAACATTATGGCTAAGAAGATTTTCACCTCTGCGCTGGGTACCc    >  1:159826/1‑300`  
                                                                                                                                                                                                                                                                                                            `GACTATGTCAAACGTAAATACAGGTTCACTTAGTGTGGACAATAAGAAGTTTTGGGCTACCGTAGAGTCCTCGGAGCATTCCTTCGAGGTTCCAATCTACGCTGAGACCCTAGACGAAGCTCTGGAGTTAGCCGAATGGCAATACGTTCCGGCTGGCTTTGAGGTTACTCGTGTGCGTCCTTGTGTAGCACCGAAGTAATACGACTCACTATTAGGGAAGACTCCCTCTGAGAAACCAAACGAAACCTAAAGGAGATTAACATTATGGCTAAGAAGATTTTCACCTCTGCGCTGGGTACCG    >  1:267221/1‑301`  
                                                                                                                                                                                                                                                                                                            `GACTATGTCAAACGTAAATACAGGTTCACTTAGTGTGGACAATAAGAAGTTTTGGGCTACCGTAGAGTCCTCGGAGCATTCCTTCGAGGTTCCAATCTACGCTGAGACCCTAGACGAAGCTCTGGAGTTAGCCGAATGGCAATACGTTCCGGCTGGCTTTGAGGTTACTCGTGTGCGTCCTTGTGTAGCACCGAAGTAATACGACTCACTATTAGGGAAGACTCCCTCTGAGAAACACAACGAAACCTAAAGGAGATTAACATTATGGCTAAGAAGATTTTCACCTCTGGGCTGGGTACCG    >  1:91078/1‑301`  
                                                                                                                                                                                                                                                                                                            `GACTATGTCAAACGTAAATACAGGTTCACTTAGTGTGGACAATAAGAAGTTTTGGGCTACCGTAGAGTCCTCGGAGCATTCCTTCGAGGTTCCAATCTACGCTGAGACCCTAGACGAAGCTCTGGAGTTAGCCGAATGGCAATACGTTCCGGCTGGCTTTGAGGTTACTCGTGTGCGTCCTCGTGTAGCACCGAAGTAATACGACTCACTATTAGGGAAGACTCCCTCTGAGAAACCAAACGAAACCTAAAGGAGATTAACATTATGGCTAAGAAGATTTTCACCTCTGCGCTGGGTCCCt    >  1:329178/1‑300`  
                                                                                                                                                                                                                                                                                                             `ACTATGTCAAAGGTAAATAGAGGTTCACGTAGGGTGGAAAATAAGAAGTTTTGGGCGACCGTAGAGTCCTCGGAGCATTCCTTCGAGGTTCCTATCTACGCTGATACCCGAAACGAAGCTCTGGAGTTAGCCGAATGGCAATACGTTCCGGCTGGCTTTGAGGTTACTCGTATGCGTCCTTGTGTAGCACCGAAGTAATACGACTCATTATTAGGGAAGACTCCCTCTGAGAAACCAAACGAAACCTAAAGGAGATTAACATTATGGCTAAGAAGATTTTCACCTCTGCGCTGGGTACCGC   <  1:230332/301‑1`  
                                                                                                                                                                                                                                                                                                             `ACTATGTCAAACGTAAATACAGGTTCACTTAGTGTGGACAATAAGAAGTTTTGGGCTACCGTAGAGTCCTCGGAGCATTCCTTCGAGGTTCCAATCTACGCTGAGACCCTAGACGAAGCTCTGGAGTTAGCCGAATGGCAATACGTTCCGGCTGGCTTTGAGGTTACTCGTGTGCGTCCTTGTGTAGCACCGAAGTAATACGACTCACTATTAGGGAAGACTCCCTCTGAGAAACCAAACGAAACCTAACGGAGATTAACATTATGGCTAAGAAGATTTTCACCTCTGCGCTGGGTACCGC   >  1:122951/1‑301`  
                                                                                                                                                                                                                                                                                                             `ACTATGTCAAACGTAAATACAGGTTCACTTAGTGTGGACAATAAGAAGTTTTGGGCTACCGTAGAGTCCTCGGAGCATTCCTTCGAGGTTCCAATCTACGCTGAGACCCTAGACGAAGCTCTGGAGTTAGCCGAATGGCAATACGTTCCGGCTGGCTTTGAGGTTACTCGTGTGCGTCCTTGTGTAGCACCGAAGTAATACGACTCACTATTAGGGAAGACTCCCTCTGAGAAACCAAACGAAACCTAACGGAGATTAACATTATGGCTAAGAAGATTTTCACCTCTGCGCTGGGTACCGC   <  1:137076/301‑1`  
                                                                                                                                                                                                                                                                                                             `ACTATGTCAAACGTAAATACAGGTTCACTTAGTGTGGACAATAAGAAGTTTTGGGCTACCGTAGAGTCCTCGGAGCATTCCTTCGAGGTTCCAATCTACGCTGAGACCCTAGACGAAGCTCTGGAGTTAGCCGAATGGCAATACGTTCCGGCTGGCTTTGAGGTTACTCGTGTGCGTCCTTGTGTAGCACCGAAGTAATACGACTCACTATTAGGGAAGACTCCCTCTGAGAAACCAAACGAAACCTAACGGAGATTAACATTATGGCTAAGAAGATTTTCACCTCTGCGCTGGGTACCGC   <  1:50815/301‑1`  
                                                                                                                                                                                                                                                                                                             `ACTATGTCAAACGTAAATACAGGTTCACTTAGTGTGGACAATAAGAAGTTTTGGGCTACCGTAGAGTCCTCGGAGCATTCCTTCGAGGTTCCAATCTACGCTGAGACCCTAGACGAAGCTCTGGAGTTAGCCGAATGGCAATACGTTCCGGCTGGCTTTGAGGTTACTCGTGTGCGTCCTTGTGTAGCACCGAAGTAATACGACTCACTATTAGGGAAGACTCCCTCTGAGAAACCAAACGAAACCTAAAGGAGATTAACATTATGGCTAAGAAGATTTTCACCTCTGCGCTGGGTACCGC   <  1:244121/301‑1`  
                                                                                                                                                                                                                                                                                                             `ACTATGTCAAACGTAAATACAGGTTCACTTAGTGTGGACAATAAGAAGTTTTGGGCTACCGTAGAGTCCTCGGAGCATTCCTTCGAGGTTCCAATCTACGCTGAGACCCTACACGAAGCTCTGGAGTTAGCCGAATGGCAATACGTTCCGGCTGGCTTTGAGGTTACTCGTGTGCGTCCTTGTGTAGCACCGAAGTAATACGACTCACTATTAGGGAAGACTCCCTCTGAGAAACCAAACGAAACCTAAAGGAGATTAACATTATGGCTAAGAAGATTTTCACCTCTGCGCTGGGTACCGC   <  1:118739/301‑1`  
                                                                                                                                                                                                                                                                                                             `ACTATGTCAAACGTAAATACAGGTTCACTTAGTGTGGACAATAAGAAGTTGTGGGCTACCGTAGAGTCCTCGGAGCATTCCTTCGAGGTGCCAATCTACGCTGAGACCCTAGACGAAGCTCTGGAGTTAGCCGAATGGCAATACGTTCCGGCTGGCTTTGAGGTTACTCGTGTGCGTCCTTGTGTAGCACCGAAGTAATACGACTCACTATTAGGGAAGACTCCCTCTGAGAAACCAAACGAAACCTAAAGGAGATTAACATTATGGCTAAGAAGATTTTCACCTCTGCGCTGGGTACCGC   <  1:49369/301‑1`  
                                                                                                                                                                                                                                                                                                             `ACTATGTCAAACGTAAATACAGGTTCACTTAGTGTGAACAATAAGAAGTTTTGGGCTACCGTAGAGTCCTCGGAGCATTCCTTCGAGGTTCCAATCTACGCTGAGACCCTAGACGAAGCTCTGGAGTTAGCCGAATGGCAATACGTTCCGGCTGGCTTTGAGGTTACTCGTGTGCGTCCTTGTGTAGCACCGAAGTAATACGACTCACTATTAGGGAAGACTCCCTCTGAGAAACCAAACGAAACCTAAAGGAGATTAACATTATGGCTAAGAAGATTTTCACCTCTGCGCTGGGTACCGC   <  1:248251/301‑1`  
                                                                                                                                                                                                                                                                                                             `ACTATGTCAAACGTAAATACAGGGTCACTTAGTGTGGACAATAAGAAGTTTTGGGCTACCGTAGAGTCCTCGGAGCATTCCTTCGAGGTTCCAATCTACGCTGAGACCCTAGACGAAGCTCTGGAGTTAGCCGAATGGCAATACGTTCCGGCTGGCTTTGAGGTTACTCGTGTGCGTCCTTGTGTAGCACCGAAGTAATACGACTCACTATTAGGGAAGACTCCCTCTGAGAAACCAAACGAAACCTAAAGGAGATTAACATTATGGCTAAGAAGATTTTCACCTCTGCGCTGGGTACCGC   <  1:87168/301‑1`  
                                                                                                                                                                                                                                                                                                             `ACTAGGTCAAACGTAAATACAGGTTCACTTAGTGTGGACAATAAGAAGTTTTGGGCTACCGTAGAGTCCTCGGAGAATTCCTTCGAGGTTCCAATCTACGCTGAGACCCTAGACGAAGCTCTGGAGTTAGCCGAATGGCAATACGTTCCGGCTGGCTTTGAGGTTACTCGTGTGCGTCCTTGTGTAGCACCGAAGTAATACGACTCACTATTAGGGAAGACTCCCTCTGAGAAACCAAACGAAACCTAAAGGAGATTAACATTATGGCTAAGAAGATTTTCACCTCTGCGCTGGGTACCGC   <  1:177602/301‑1`  
                                                                                                                                                                                                                                                                                                              `CTATGTCAAACGTAAATACAGGTTCACTTAGTGTGGACAATAAGAAGTTTTGGGCTACCGTAGAGTCCTCGGAGCATTCCTTCGAGGTTCCAATCTACGCTGAGACCCTAGACGAAGCTCTGGAGTTAGCCGAATGGCAATACGTTCCGGCTGGCTTTGAGGTTACTCGTGTGCGTCCTTGTGTAGCACCGAAGTAATACGACTCACTATTAGGGAAGACTCCCTCTGAGAAACCAAACGAAACCTAAAGGAGATTAACATTATGGCTAAGAAGGTTTTCACCTCTGCGCTGGGTACCGCT  >  1:50798/1‑301`  
                                                                                                                                                                                                                                                                                                              `CTATGTCAAACGTAAATACAGGTTCACTTAGTGTGGACAATAAGAAGTTTTGGGCTACCGTAGAGTCCTCGGAGCATTCCTTCGAGGTTCCAATCTACGCTGAGACCCTAGACGAAGCTCTGGAGTTAGCCGAATGGCAATACGTTCCGGCTGGCTTTGAGGTTACTCGTGTGCGTCCTTGTGTAGCACCGAAGTAATACGACTCACTATTAGGGAAGACTCCCTCTGAGAAACCAAACGAAACCTAAAGGAGATTAACATTATGGCTAAGAAGATTTTCACCTCTGCGCTGGGTACCGCT  >  1:328282/1‑301`  
                                                                                                                                                                                                                                                                                                              `CTATGTCAAACGTAAATACAGGTTCACTTAGTGTGGACAATAAGAAGTTTTGGGCTACCGTAGAGTCCTCGGAGCATTCCTTCGAGGTTCCAATCTACGCTGAGACCCTAGACGAAGCTCTGGAGTTAGCCGAATGGCAATACGTTCCGGCTGGCTTTGAGGTTACTCGTGTGCGTCCTTGTGTAGCACCGAAGTAATACGACTCACTATTAGGGAAGACTCCCTCTGAGAAACCAAACGAAACCTAAAGGAGATTAACATTATGGCTAAGAAGATTTTCACCTCTGCGCGGGGTACCGCT  >  1:339937/1‑301`  
                                                                                                                                                                                                                                                                                                              `CTATGTCAAACGTAAATACAGGTTCACTTAGTGTGGACAATAAGAAGTTTTGGGCTACCGTAGAGTCCTCGGAGCATTCCTTCGAGGTTCCAATCTACGCTGAGACCCTAGACGAAGCTCTGGAGTTAGCCGAATGGCAATACGTTCAGGCTGGTTTTGAGGTTACTCGTGTGCGTCCTTGTGTAGCACCGAAGTAATACGACTCACTATTAGGGAAGACTCCCCCAGAGAAACCAAACGAAACCTAAATGAGATCAACATTATGGCAAAGAAGATTTTCACCTCTGCGCTGGGTACCGCT  >  1:151935/1‑301`  
                                                                                                                                                                                                                                                                                                              `CTATGTCAAACGTAAATACAGGTTCACTTAGTGTGGACAATAAGAAGTTTTGGGCTACCGTAGAGTCCTCGGAGCATTCCTTCGAGGTTCCAATCTACGCTGAGACCCTAGACGAAGCTCTGGAGTTAGCCGAATGACAATACGTTCCGGCTGGCTTTGAGGTTACTCGTGTGCGTCCTTGTGTAGCACCGAAGTAATACGACTCACTATTAGGGAAGACTCCCTCTGAGAAACCAAACGAAACCTAAAGGAGATTAACATTATGGCTAAGAAGATTTTCACCTCTGCGCTGGGGACCGCT  >  1:188306/1‑301`  
                                                                                                                                                                                                                                                                                                              `CTATGTCAAACGTAAATACAGGTTCACTTAGTGTGGACAATAAGAAGTTTTGGGCTACCGTAGAGTCCTCGGAGCATTCCTTCGAGGTTCCAATCCACGCTGAGACCCTAGACGAAGCTCTGGAGTTAGCCGAATGGCAATACGTTCCGGCTGGCTTTGAGGTTACTCGTGTGCGTCCTTGTGTAGCACCGAAGTAATACGACTCACTATTAGGGAAGACTCCCTCTGAGAAACCAAACGAAACCTAAAGGAGATTAACATTATGGCTAAGAAGATTTTCACCTCTGCGCTGGGTACCGCT  >  1:138486/1‑301`  
                                                                                                                                                                                                                                                                                                              `CTATGTCAAACGTAAATACAGGTTCACTTAGTGTGGACAATAAGAAGTTTTGGGCTACCGTAGAGTCCTCGGAGCATTCCTTCGAGGTTCAAATCTACGCTGAGACCCTAGACGAAGCTCTGGAGTTAGCCGAATGGCAATACGTTCCGGCTGGCTTTGAGGTTACTCGTGTGCGTCCTTGTGTAGCACCGAAGTAATACGACTCACTATTAGGGAAGACTCCCTCTGAGAAACCAAACGAAACCTAAAGGAGATTAACATTATGGCTAAGAAGATTTTCACCTCTGCGCTGGGTACCGat  >  1:300162/1‑299`  

                                                                                                                                                                                                                                                                                                                                                                                                                                                                                                                                                                                                                             
`ACCCAGCGCAGAGGTGAAAATCTTCTTAGCCATAATGTTAATCTCCTTTAGGTTTCGTTTGGTTTCTCAGAGGGAGTCTTCCCTAATAGTGAGTCGTATTACTTCGGTGCTACACAAGGACGCACACGAGTAACCTCAAAGCCAGCCGGAACGTATTGCCATTCGGCTAACTCCAGAGCTTCGTCTAGGGTCTCAGCGTAGATTGGAACCTCGAAGGAATGCTCCGAGGACTCTACGGTAGCCCAAAACTTCTTATTGTCCACACTAAGTGAACCTGTATTTACGTTTGACATAGTCATTGAC‑‑‑‑‑‑‑‑‑‑‑‑‑‑‑‑‑‑‑‑‑‑‑‑‑‑‑‑‑‑‑‑‑‑‑‑‑‑‑‑‑‑‑‑‑‑‑‑‑‑‑‑‑‑‑‑‑‑‑‑‑‑‑‑‑‑‑‑‑‑‑‑‑‑‑‑‑‑‑‑‑‑‑‑‑‑‑‑‑‑‑‑‑‑‑‑‑‑‑‑‑‑‑‑‑‑‑‑‑‑‑‑‑‑‑‑‑‑‑‑‑‑‑‑‑‑‑‑‑‑‑‑‑‑‑‑‑‑‑‑‑‑‑‑‑‑‑‑‑‑‑‑‑‑‑‑‑‑‑‑‑‑‑‑‑‑‑‑‑‑‑‑‑‑‑‑‑‑‑‑‑‑‑‑‑‑‑‑‑‑‑‑‑‑‑‑‑‑‑‑‑‑‑‑‑‑‑‑‑‑‑‑‑‑‑‑‑‑‑‑‑‑‑‑‑‑‑‑‑‑‑‑‑‑‑‑‑‑‑‑‑‑‑‑‑‑‑‑‑‑‑‑‑‑‑‑‑‑‑‑‑‑‑‑‑‑‑‑‑‑‑‑‑‑‑‑‑‑‑‑‑‑‑‑‑‑‑‑‑‑‑‑‑‑‑‑‑‑‑‑  <  NC_001604.1/9190‑8888`  
`‑‑‑‑‑‑‑‑‑‑‑‑‑‑‑‑‑‑‑‑‑‑‑‑‑‑‑‑‑‑‑‑‑‑‑‑‑‑‑‑‑‑‑‑‑‑‑‑‑‑‑‑‑‑‑‑‑‑‑‑‑‑‑‑‑‑‑‑‑‑‑‑‑‑‑‑‑‑‑‑‑‑‑‑‑‑‑‑‑‑‑‑‑‑‑‑‑‑‑‑‑‑‑‑‑‑‑‑‑‑‑‑‑‑‑‑‑‑‑‑‑‑‑‑‑‑‑‑‑‑‑‑‑‑‑‑‑‑‑‑‑‑‑‑‑‑‑‑‑‑‑‑‑‑‑‑‑‑‑‑‑‑‑‑‑‑‑‑‑‑‑‑‑‑‑‑‑‑‑‑‑‑‑‑‑‑‑‑‑‑‑‑‑‑‑‑‑‑‑‑‑‑‑‑‑‑‑‑‑‑‑‑‑‑‑‑‑‑‑‑‑‑‑‑‑‑‑‑‑‑‑‑‑‑‑‑‑‑‑‑‑‑‑‑‑‑‑‑‑‑‑‑‑‑‑‑‑‑‑‑‑‑‑‑‑‑‑‑‑‑‑‑‑‑‑‑‑‑‑‑‑‑‑‑‑‑‑‑‑‑‑‑‑‑‑‑‑‑‑tgacTATGTCAAACGTAAATACAGGTTCACTTAGTGTGGACAATAAGAAGTTTTGGGCTACCGTAGAGTCCTCGGAGCATTCCTTCGAGGTTCCAATCTACGCTGAGACCCTAGACGAAGCTCTGGAGTTAGCCGAATGGCAATACGTTCCGGCTGGCTTTGAGGTTACTCGTGTGCGTCCTTGTGTAGCACCGAAGTAATACGACTCACTATTAGGGAAGACTCCCTCTGAGAAACCAAACGAAACCTAAAGGAGATTAACATTATGGCTAAGAAGATTTTCACCTCTGCGCTGGGTACCGCT  >  NC_001604.1/8897‑9196`

  

Alignment Legend

Aligned base mismatch/match (shaded by quality score): `ATCG/ATCG` `< 3 ≤` `ATCG/ATCG` `< 11 ≤` `ATCG/ATCG` `< 24 ≤` `ATCG/ATCG` `< 35 ≤` `ATCG/ATCG` `< 38 ≤` `ATCG/ATCG`

Unaligned base: `atcg`    Masked matching base: `atcg`    Alignment gap: `‑`    Deleted base: `‑`

Reads not counted as support for junction

read\_name Not counted due to insufficient overlap past the breakpoint.

read\_name Not counted due to not crossing MOB target site duplication.
